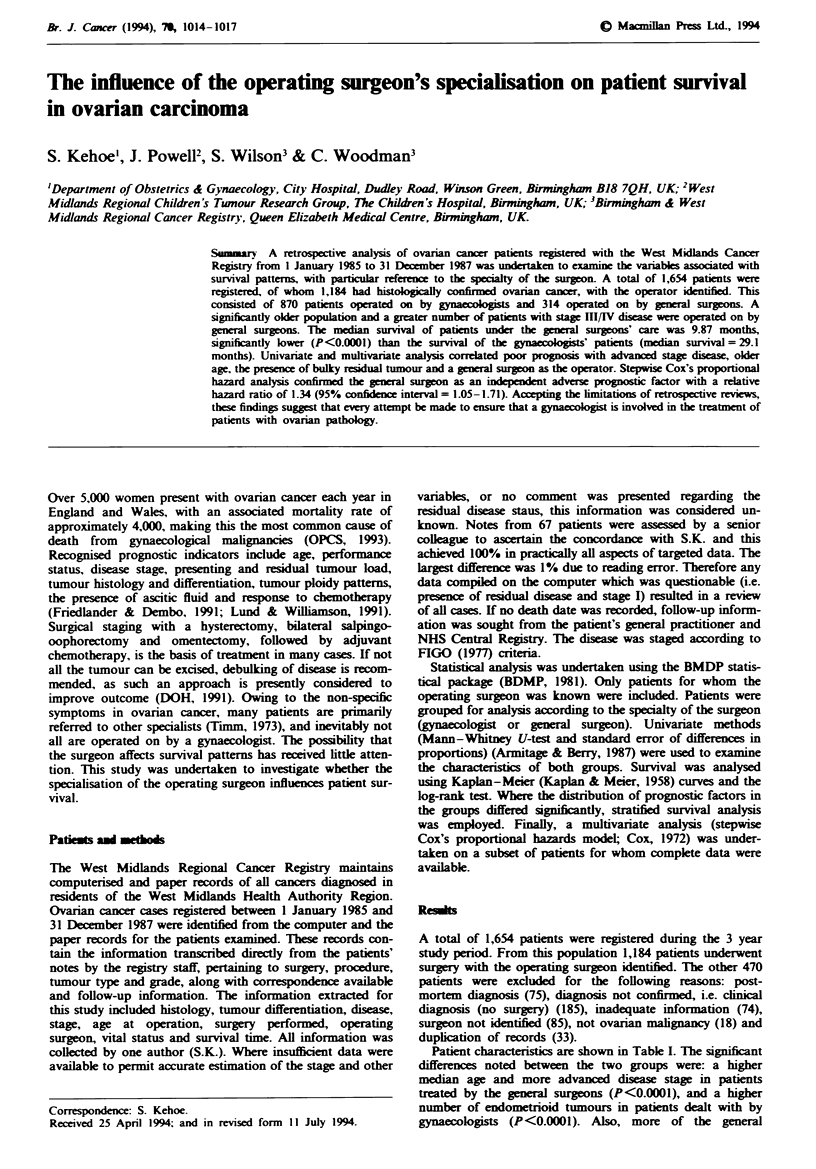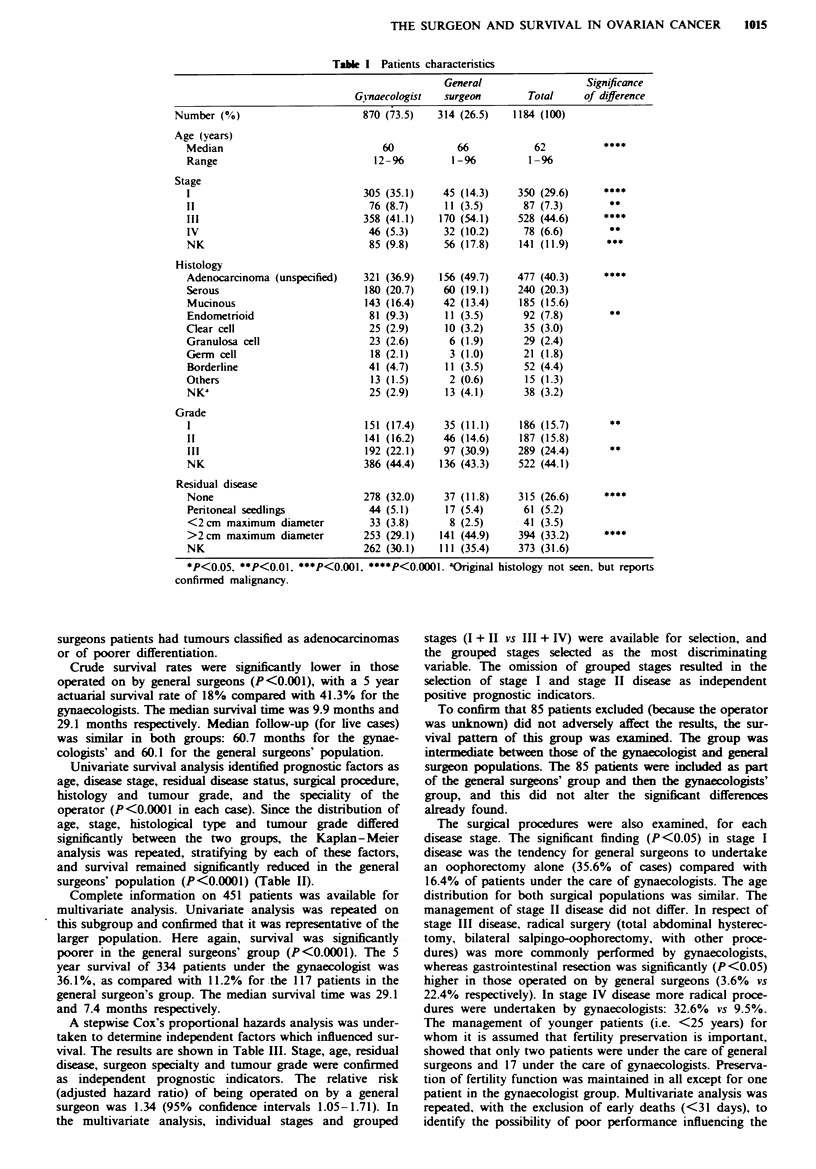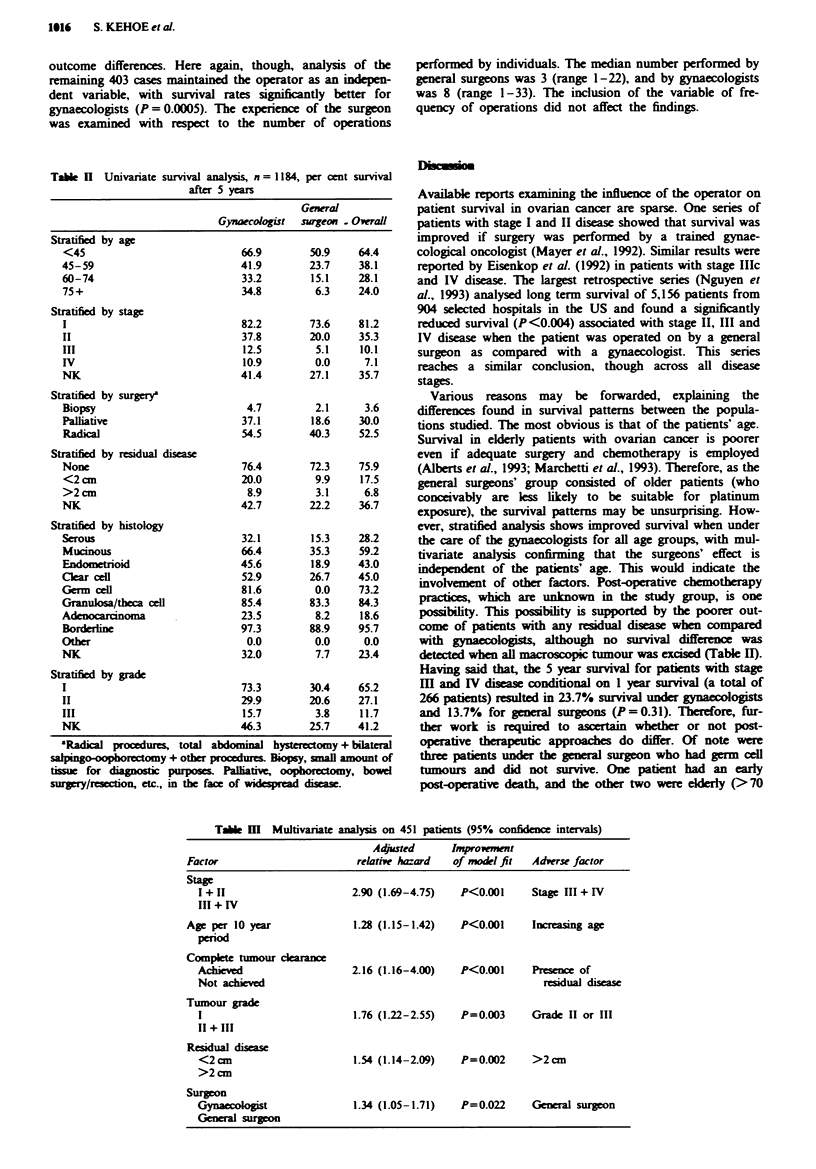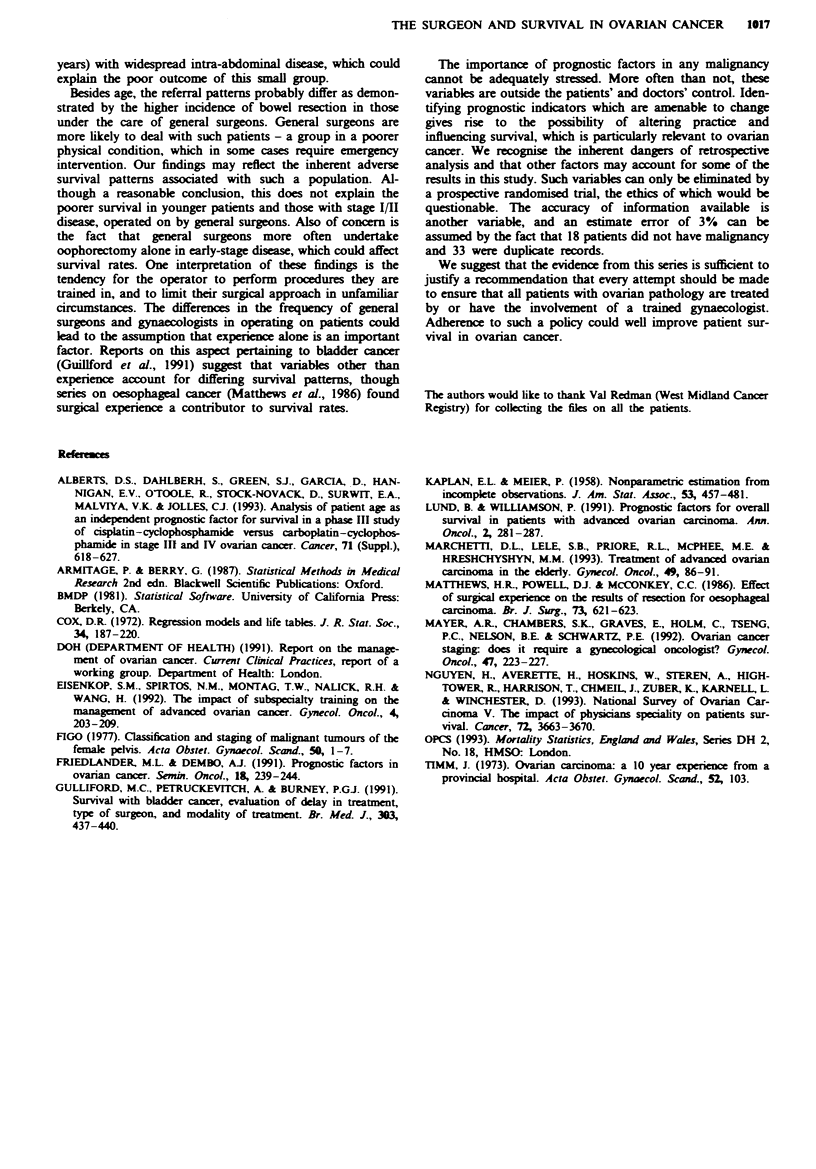# The influence of the operating surgeon's specialisation on patient survival in ovarian carcinoma.

**DOI:** 10.1038/bjc.1994.440

**Published:** 1994-11

**Authors:** S. Kehoe, J. Powell, S. Wilson, C. Woodman

**Affiliations:** Department of Obstetrics & Gynaecology, City Hospital, Birmingham, UK.

## Abstract

A retrospective analysis of ovarian cancer patients registered with the West Midlands Cancer Registry from 1 January 1985 to 31 December 1987 was undertaken to examine the variables associated with survival patterns, with particular reference to the specialty of the surgeon. A total of 1,654 patients were registered, of whom 1,184 had histologically confirmed ovarian cancer, with the operator identified. This consisted of 870 patients operated on by gynaecologists and 314 operated on by general surgeons. A significantly older population and a greater number of patients with stage III/IV disease were operated on by general surgeons. The median survival of patients under the general surgeons' care was 9.87 months, significantly lower (P < 0.0001) than the survival of the gynaecologists' patients (median survival = 29.1 months). Univariate and multivariate analysis correlated poor prognosis with advanced stage disease, older age, the presence of bulky residual tumour and a general surgeon as the operator. Stepwise Cox's proportional hazard analysis confirmed the general surgeon as an independent adverse prognostic factor with a relative hazard ratio of 1.34 (95% confidence interval = 1.05-1.71). Accepting the limitations of retrospective reviews, these findings suggest that every attempt be made to ensure that a gynaecologist is involved in the treatment of patients with ovarian pathology.


Br. J. Cancer (1994), 70, 1014-1017 © Macmillan Press Ltd., 1994

### The influence of the operating surgeon's specialisation on patient survival in ovarian carcinoma

S. Kehoe[1], J. Powell[2], S. Wilson[3] & C. Woodman[3]

[1]*Department of Obstetrics & Gynaecology, City Hospital, Dudley Road, Winson Green, Birmingham B18 7QH, UK;* [2]*West Midlands Regional Children's Tumour Research Group, The Children's Hospital, Birmingham, UK;* [3]*Birmingham & West Midlands Regional Cancer Registry, Queen Elizabeth Medical Centre, Birmingham, UK.*

**Summary** A retrospective analysis of ovarian cancer patients registered with the West Midlands Cancer Registry from 1 January 1985 to 31 December 1987 was undertaken to examine the variables associated with survival patterns, with particular reference to the specialty of the surgeon. A total of 1,654 patients were registered, of whom 1,184 had histologically confirmed ovarian cancer, with the operator identified. This consisted of 870 patients operated on by gynaecologists and 314 operated on by general surgeons. A significantly older population and a greater number of patients with stage III/IV disease were operated on by general surgeons. The median survival of patients under the general surgeons' care was 9.87 months, significantly lower ($P<0.0001$) than the survival of the gynaecologists' patients (median survival = 29.1 months). Univariate and multivariate analysis correlated poor prognosis with advanced stage disease, older age, the presence of bulky residual tumour and a general surgeon as the operator. Stepwise Cox's proportional hazard analysis confirmed the general surgeon as an independent adverse prognostic factor with a relative hazard ratio of 1.34 (95% confidence interval = 1.05-1.71). Accepting the limitations of retrospective reviews, these findings suggest that every attempt be made to ensure that a gynaecologist is involved in the treatment of patients with ovarian pathology.

Over 5,000 women present with ovarian cancer each year in England and Wales, with an associated mortality rate of approximately 4,000, making this the most common cause of death from gynaecological malignancies (OPCS, 1993). Recognised prognostic indicators include age, performance status, disease stage, presenting and residual tumour load, tumour histology and differentiation, tumour ploidy patterns, the presence of ascitic fluid and response to chemotherapy (Friedlander & Dembo, 1991; Lund & Williamson, 1991). Surgical staging with a hysterectomy, bilateral salpingo-oophorectomy and omentectomy, followed by adjuvant chemotherapy, is the basis of treatment in many cases. If not all the tumour can be excised, debulking of disease is recommended, as such an approach is presently considered to improve outcome (DOH, 1991). Owing to the non-specific symptoms in ovarian cancer, many patients are primarily referred to other specialists (Timm, 1973), and inevitably not all are operated on by a gynaecologist. The possibility that the surgeon affects survival patterns has received little attention. This study was undertaken to investigate whether the specialisation of the operating surgeon influences patient survival.

### Patients and methods

The West Midlands Regional Cancer Registry maintains computerised and paper records of all cancers diagnosed in residents of the West Midlands Health Authority Region. Ovarian cancer cases registered between 1 January 1985 and 31 December 1987 were identified from the computer and the paper records for the patients examined. These records contain the information transcribed directly from the patients' notes by the registry staff, pertaining to surgery, procedure, tumour type and grade, along with correspondence available and follow-up information. The information extracted for this study included histology, tumour differentiation, disease, stage, age at operation, surgery performed, operating surgeon, vital status and survival time. All information was collected by one author (S.K.). Where insufficient data were available to permit accurate estimation of the stage and other variables, or no comment was presented regarding the residual disease staus, this information was considered unknown. Notes from 67 patients were assessed by a senior colleague to ascertain the concordance with S.K. and this achieved 100% in practically all aspects of targeted data. The largest difference was 1% due to reading error. Therefore any data compiled on the computer which was questionable (i.e. presence of residual disease and stage I) resulted in a review of all cases. If no death date was recorded, follow-up information was sought from the patient's general practitioner and NHS Central Registry. The disease was staged according to FIGO (1977) criteria.

Statistical analysis was undertaken using the BMDP statistical package (BDMP, 1981). Only patients for whom the operating surgeon was known were included. Patients were grouped for analysis according to the specialty of the surgeon (gynaecologist or general surgeon). Univariate methods (Mann-Whitney *U*-test and standard error of differences in proportions) (Armitage & Berry, 1987) were used to examine the characteristics of both groups. Survival was analysed using Kaplan-Meier (Kaplan & Meier, 1958) curves and the log-rank test. Where the distribution of prognostic factors in the groups differed significantly, stratified survival analysis was employed. Finally, a multivariate analysis (stepwise Cox's proportional hazards model; Cox, 1972) was undertaken on a subset of patients for whom complete data were available.

### Results

A total of 1,654 patients were registered during the 3 year study period. From this population 1,184 patients underwent surgery with the operating surgeon identified. The other 470 patients were excluded for the following reasons: post-mortem diagnosis (75), diagnosis not confirmed, i.e. clinical diagnosis (no surgery) (185), inadequate information (74), surgeon not identified (85), not ovarian malignancy (18) and duplication of records (33).

Patient characteristics are shown in Table I. The significant differences noted between the two groups were: a higher median age and more advanced disease stage in patients treated by the general surgeons ($P<0.0001$), and a higher number of endometrioid tumours in patients dealt with by gynaecologists ($P<0.0001$). Also, more of the general

Correspondence: S. Kehoe.
Received 25 April 1994; and in revised form 11 July 1994.

**Table I** Patients characteristics

| | *Gynaecologist* | *General surgeon* | *Total* | *Significance of difference* |
|---|---|---|---|---|
| Number (%) | 870 (73.5) | 314 (26.5) | 1184 (100) | |
| Age (years) | | | | |
| Median | 60 | 66 | 62 | **** |
| Range | 12-96 | 1-96 | 1-96 | |
| Stage | | | | |
| I | 305 (35.1) | 45 (14.3) | 350 (29.6) | **** |
| II | 76 (8.7) | 11 (3.5) | 87 (7.3) | ** |
| III | 358 (41.1) | 170 (54.1) | 528 (44.6) | **** |
| IV | 46 (5.3) | 32 (10.2) | 78 (6.6) | ** |
| NK | 85 (9.8) | 56 (17.8) | 141 (11.9) | *** |
| Histology | | | | |
| Adenocarcinoma (unspecified) | 321 (36.9) | 156 (49.7) | 477 (40.3) | **** |
| Serous | 180 (20.7) | 60 (19.1) | 240 (20.3) | |
| Mucinous | 143 (16.4) | 42 (13.4) | 185 (15.6) | |
| Endometrioid | 81 (9.3) | 11 (3.5) | 92 (7.8) | ** |
| Clear cell | 25 (2.9) | 10 (3.2) | 35 (3.0) | |
| Granulosa cell | 23 (2.6) | 6 (1.9) | 29 (2.4) | |
| Germ cell | 18 (2.1) | 3 (1.0) | 21 (1.8) | |
| Borderline | 41 (4.7) | 11 (3.5) | 52 (4.4) | |
| Others | 13 (1.5) | 2 (0.6) | 15 (1.3) | |
| NK[a] | 25 (2.9) | 13 (4.1) | 38 (3.2) | |
| Grade | | | | |
| I | 151 (17.4) | 35 (11.1) | 186 (15.7) | ** |
| II | 141 (16.2) | 46 (14.6) | 187 (15.8) | |
| III | 192 (22.1) | 97 (30.9) | 289 (24.4) | ** |
| NK | 386 (44.4) | 136 (43.3) | 522 (44.1) | |
| Residual disease | | | | |
| None | 278 (32.0) | 37 (11.8) | 315 (26.6) | **** |
| Peritoneal seedlings | 44 (5.1) | 17 (5.4) | 61 (5.2) | |
| <2 cm maximum diameter | 33 (3.8) | 8 (2.5) | 41 (3.5) | |
| >2 cm maximum diameter | 253 (29.1) | 141 (44.9) | 394 (33.2) | **** |
| NK | 262 (30.1) | 111 (35.4) | 373 (31.6) | |

*$P<0.05$, **$P<0.01$, ***$P<0.001$, ****$P<0.0001$. [a]Original histology not seen, but reports confirmed malignancy.

surgeons patients had tumours classified as adenocarcinomas or of poorer differentiation.

Crude survival rates were significantly lower in those operated on by general surgeons ($P<0.001$), with a 5 year actuarial survival rate of 18% compared with 41.3% for the gynaecologists. The median survival time was 9.9 months and 29.1 months respectively. Median follow-up (for live cases) was similar in both groups: 60.7 months for the gynaecologists' and 60.1 for the general surgeons' population.

Univariate survival analysis identified prognostic factors as age, disease stage, residual disease status, surgical procedure, histology and tumour grade, and the speciality of the operator ($P<0.0001$ in each case). Since the distribution of age, stage, histological type and tumour grade differed significantly between the two groups, the Kaplan-Meier analysis was repeated, stratifying by each of these factors, and survival remained significantly reduced in the general surgeons' population ($P<0.0001$) (Table II).

Complete information on 451 patients was available for multivariate analysis. Univariate analysis was repeated on this subgroup and confirmed that it was representative of the larger population. Here again, survival was significantly poorer in the general surgeons' group ($P<0.0001$). The 5 year survival of 334 patients under the gynaecologist was 36.1%, as compared with 11.2% for the 117 patients in the general surgeon's group. The median survival time was 29.1 and 7.4 months respectively.

A stepwise Cox's proportional hazards analysis was undertaken to determine independent factors which influenced survival. The results are shown in Table III. Stage, age, residual disease, surgeon specialty and tumour grade were confirmed as independent prognostic indicators. The relative risk (adjusted hazard ratio) of being operated on by a general surgeon was 1.34 (95% confidence intervals 1.05-1.71). In the multivariate analysis, individual stages and grouped stages (I + II *vs* III + IV) were available for selection, and the grouped stages selected as the most discriminating variable. The omission of grouped stages resulted in the selection of stage I and stage II disease as independent positive prognostic indicators.

To confirm that 85 patients excluded (because the operator was unknown) did not adversely affect the results, the survival pattern of this group was examined. The group was intermediate between those of the gynaecologist and general surgeon populations. The 85 patients were included as part of the general surgeons' group and then the gynaecologists' group, and this did not alter the significant differences already found.

The surgical procedures were also examined, for each disease stage. The significant finding ($P<0.05$) in stage I disease was the tendency for general surgeons to undertake an oophorectomy alone (35.6% of cases) compared with 16.4% of patients under the care of gynaecologists. The age distribution for both surgical populations was similar. The management of stage II disease did not differ. In respect of stage III disease, radical surgery (total abdominal hysterectomy, bilateral salpingo-oophorectomy, with other procedures) was more commonly performed by gynaecologists, whereas gastrointestinal resection was significantly ($P<0.05$) higher in those operated on by general surgeons (3.6% *vs* 22.4% respectively). In stage IV disease more radical procedures were undertaken by gynaecologists: 32.6% *vs* 9.5%. The management of younger patients (i.e. <25 years) for whom it is assumed that fertility preservation is important, showed that only two patients were under the care of general surgeons and 17 under the care of gynaecologists. Preservation of fertility function was maintained in all except for one patient in the gynaecologist group. Multivariate analysis was repeated, with the exclusion of early deaths (<31 days), to identify the possibility of poor performance influencing the outcome differences. Here again, though, analysis of the remaining 403 cases maintained the operator as an independent variable, with survival rates significantly better for gynaecologists ($P = 0.0005$). The experience of the surgeon was examined with respect to the number of operations performed by individuals. The median number performed by general surgeons was 3 (range 1–22), and by gynaecologists was 8 (range 1–33). The inclusion of the variable of frequency of operations did not affect the findings.

**Table II** Univariate survival analysis, $n = 1184$, per cent survival after 5 years

| | *Gynaecologist* | *General surgeon* | *Overall* |
|---|---|---|---|
| Stratified by age | | | |
| <45 | 66.9 | 50.9 | 64.4 |
| 45–59 | 41.9 | 23.7 | 38.1 |
| 60–74 | 33.2 | 15.1 | 28.1 |
| 75+ | 34.8 | 6.3 | 24.0 |
| Stratified by stage | | | |
| I | 82.2 | 73.6 | 81.2 |
| II | 37.8 | 20.0 | 35.3 |
| III | 12.5 | 5.1 | 10.1 |
| IV | 10.9 | 0.0 | 7.1 |
| NK | 41.4 | 27.1 | 35.7 |
| Stratified by surgery[a] | | | |
| Biopsy | 4.7 | 2.1 | 3.6 |
| Palliative | 37.1 | 18.6 | 30.0 |
| Radical | 54.5 | 40.3 | 52.5 |
| Stratified by residual disease | | | |
| None | 76.4 | 72.3 | 75.9 |
| <2 cm | 20.0 | 9.9 | 17.5 |
| >2 cm | 8.9 | 3.1 | 6.8 |
| NK | 42.7 | 22.2 | 36.7 |
| Stratified by histology | | | |
| Serous | 32.1 | 15.3 | 28.2 |
| Mucinous | 66.4 | 35.3 | 59.2 |
| Endometrioid | 45.6 | 18.9 | 43.0 |
| Clear cell | 52.9 | 26.7 | 45.0 |
| Germ cell | 81.6 | 0.0 | 73.2 |
| Granulosa/theca cell | 85.4 | 83.3 | 84.3 |
| Adenocarcinoma | 23.5 | 8.2 | 18.6 |
| Borderline | 97.3 | 88.9 | 95.7 |
| Other | 0.0 | 0.0 | 0.0 |
| NK | 32.0 | 7.7 | 23.4 |
| Stratified by grade | | | |
| I | 73.3 | 30.4 | 65.2 |
| II | 29.9 | 20.6 | 27.1 |
| III | 15.7 | 3.8 | 11.7 |
| NK | 46.3 | 25.7 | 41.2 |

[a]Radical procedures, total abdominal hysterectomy + bilateral salpingo-oophorectomy + other procedures. Biopsy, small amount of tissue for diagnostic purposes. Palliative, oophorectomy, bowel surgery/resection, etc., in the face of widespread disease.

### Discussion

Available reports examining the influence of the operator on patient survival in ovarian cancer are sparse. One series of patients with stage I and II disease showed that survival was improved if surgery was performed by a trained gynaecological oncologist (Mayer *et al.*, 1992). Similar results were reported by Eisenkop *et al.* (1992) in patients with stage IIIc and IV disease. The largest retrospective series (Nguyen *et al.*, 1993) analysed long term survival of 5,156 patients from 904 selected hospitals in the US and found a significantly reduced survival ($P < 0.004$) associated with stage II, III and IV disease when the patient was operated on by a general surgeon as compared with a gynaecologist. This series reaches a similar conclusion, though across all disease stages.

Various reasons may be forwarded, explaining the differences found in survival patterns between the populations studied. The most obvious is that of the patients' age. Survival in elderly patients with ovarian cancer is poorer even if adequate surgery and chemotherapy is employed (Alberts *et al.*, 1993; Marchetti *et al.*, 1993). Therefore, as the general surgeons' group consisted of older patients (who conceivably are less likely to be suitable for platinum exposure), the survival patterns may be unsurprising. However, stratified analysis shows improved survival when under the care of the gynaecologists for all age groups, with multivariate analysis confirming that the surgeons' effect is independent of the patients' age. This would indicate the involvement of other factors. Post-operative chemotherapy practices, which are unknown in the study group, is one possibility. This possibility is supported by the poorer outcome of patients with any residual disease when compared with gynaecologists, although no survival difference was detected when all macroscopic tumour was excised (Table II). Having said that, the 5 year survival for patients with stage III and IV disease conditional on 1 year survival (a total of 266 patients) resulted in 23.7% survival under gynaecologists and 13.7% for general surgeons ($P = 0.31$). Therefore, further work is required to ascertain whether or not post-operative therapeutic approaches do differ. Of note were three patients under the general surgeon who had germ cell tumours and did not survive. One patient had an early post-operative death, and the other two were elderly (>70

**Table III** Multivariate analysis on 451 patients (95% confidence intervals)

| *Factor* | *Adjusted relative hazard* | *Improvement of model fit* | *Adverse factor* |
|---|---|---|---|
| Stage | | | |
| I + II | 2.90 (1.69–4.75) | $P < 0.001$ | Stage III + IV |
| III + IV | | | |
| Age per 10 year period | 1.28 (1.15–1.42) | $P < 0.001$ | Increasing age |
| Complete tumour clearance | | | |
| Achieved | 2.16 (1.16–4.00) | $P < 0.001$ | Presence of residual disease |
| Not achieved | | | |
| Tumour grade | | | |
| I | 1.76 (1.22–2.55) | $P = 0.003$ | Grade II or III |
| II + III | | | |
| Residual disease | | | |
| <2 cm | 1.54 (1.14–2.09) | $P = 0.002$ | >2 cm |
| >2 cm | | | |
| Surgeon | | | |
| Gynaecologist | 1.34 (1.05–1.71) | $P = 0.022$ | General surgeon |
| General surgeon | | | |

years) with widespread intra-abdominal disease, which could explain the poor outcome of this small group.

Besides age, the referral patterns probably differ as demonstrated by the higher incidence of bowel resection in those under the care of general surgeons. General surgeons are more likely to deal with such patients – a group in a poorer physical condition, which in some cases require emergency intervention. Our findings may reflect the inherent adverse survival patterns associated with such a population. Although a reasonable conclusion, this does not explain the poorer survival in younger patients and those with stage I/II disease, operated on by general surgeons. Also of concern is the fact that general surgeons more often undertake oophorectomy alone in early-stage disease, which could affect survival rates. One interpretation of these findings is the tendency for the operator to perform procedures they are trained in, and to limit their surgical approach in unfamiliar circumstances. The differences in the frequency of general surgeons and gynaecologists in operating on patients could lead to the assumption that experience alone is an important factor. Reports on this aspect pertaining to bladder cancer (Guillford *et al.*, 1991) suggest that variables other than experience account for differing survival patterns, though series on oesophageal cancer (Matthews *et al.*, 1986) found surgical experience a contributor to survival rates.

The importance of prognostic factors in any malignancy cannot be adequately stressed. More often than not, these variables are outside the patients' and doctors' control. Identifying prognostic indicators which are amenable to change gives rise to the possibility of altering practice and influencing survival, which is particularly relevant to ovarian cancer. We recognise the inherent dangers of retrospective analysis and that other factors may account for some of the results in this study. Such variables can only be eliminated by a prospective randomised trial, the ethics of which would be questionable. The accuracy of information available is another variable, and an estimate error of 3% can be assumed by the fact that 18 patients did not have malignancy and 33 were duplicate records.

We suggest that the evidence from this series is sufficient to justify a recommendation that every attempt should be made to ensure that all patients with ovarian pathology are treated by or have the involvement of a trained gynaecologist. Adherence to such a policy could well improve patient survival in ovarian cancer.

The authors would like to thank Val Redman (West Midland Cancer Registry) for collecting the files on all the patients.

### References

ALBERTS, D.S., DAHLBERH, S., GREEN, S.J., GARCIA, D., HANNIGAN, E.V., O'TOOLE, R., STOCK-NOVACK, D., SURWIT, E.A., MALVIYA, V.K. & JOLLES, C.J. (1993). Analysis of patient age as an independent prognostic factor for survival in a phase III study of cisplatin-cyclophosphamide versus carboplatin-cyclophosphamide in stage III and IV ovarian cancer. *Cancer*, 71 (Suppl.), 618-627.

ARMITAGE, P. & BERRY, G. (1987). *Statistical Methods in Medical Research* 2nd edn. Blackwell Scientific Publications: Oxford.

BMDP (1981). *Statistical Software*. University of California Press: Berkely, CA.

COX, D.R. (1972). Regression models and life tables. *J. R. Stat. Soc.*, **34**, 187-220.

DOH (DEPARTMENT OF HEALTH) (1991). Report on the management of ovarian cancer. *Current Clinical Practices*, report of a working group. Department of Health: London.

EISENKOP, S.M., SPIRTOS, N.M., MONTAG, T.W., NALICK, R.H. & WANG, H. (1992). The impact of subspecialty training on the management of advanced ovarian cancer. *Gynecol. Oncol.*, **4**, 203-209.

FIGO (1977). Classification and staging of malignant tumours of the female pelvis. *Acta Obstet. Gynaecol. Scand.*, **50**, 1-7.

FRIEDLANDER, M.L. & DEMBO, A.J. (1991). Prognostic factors in ovarian cancer. *Semin. Oncol.*, **18**, 239-244.

GULLIFORD, M.C., PETRUCKEVITCH, A. & BURNEY, P.G.J. (1991). Survival with bladder cancer, evaluation of delay in treatment, type of surgeon, and modality of treatment. *Br. Med. J.*, **303**, 437-440.

KAPLAN, E.L. & MEIER, P. (1958). Nonparametric estimation from incomplete observations. *J. Am. Stat. Assoc.*, **53**, 457-481.

LUND, B. & WILLIAMSON, P. (1991). Prognostic factors for overall survival in patients with advanced ovarian carcinoma. *Ann. Oncol.*, **2**, 281-287.

MARCHETTI, D.L., LELE, S.B., PRIORE, R.L., MCPHEE, M.E. & HRESHCHYSHYN, M.M. (1993). Treatment of advanced ovarian carcinoma in the elderly. *Gynecol. Oncol.*, **49**, 86-91.

MATTHEWS, H.R., POWELL, D.J. & MCCONKEY, C.C. (1986). Effect of surgical experience on the results of resection for oesophageal carcinoma. *Br. J. Surg.*, **73**, 621-623.

MAYER, A.R., CHAMBERS, S.K., GRAVES, E., HOLM, C., TSENG, P.C., NELSON, B.E. & SCHWARTZ, P.E. (1992). Ovarian cancer staging: does it require a gynecological oncologist? *Gynecol. Oncol.*, **47**, 223-227.

NGUYEN, H., AVERETTE, H., HOSKINS, W., STEREN, A., HIGHTOWER, R., HARRISON, T., CHMEIL, J., ZUBER, K., KARNELL, L. & WINCHESTER, D. (1993). National Survey of Ovarian Carcinoma V. The impact of physicians speciality on patients survival. *Cancer*, **72**, 3663-3670.

OPCS (1993). *Mortality Statistics, England and Wales*, Series DH 2, No. 18, HMSO: London.

TIMM, J. (1973). Ovarian carcinoma: a 10 year experience from a provincial hospital. *Acta Obstet. Gynaecol. Scand.*, **52**, 103.